# Strategic orientation, business model innovation and corporate performance—Evidence from construction industry

**DOI:** 10.3389/fpsyg.2022.971654

**Published:** 2022-10-20

**Authors:** Wucheng Han, Yang Zhou, Ruoyu Lu

**Affiliations:** ^1^School of Economics and Management, University of Electronic Science and Technology of China, Chengdu, China; ^2^School of Finance and Public Administration, Anhui University of Finance and Economics, Bengbu, China

**Keywords:** strategic orientation, business model innovation, corporate performance, environmental dynamism, construction industry, partial least squares structural equation modelling

## Abstract

In the highly competitive Chinese construction market, developing a strategic orientation alone fails to maintain the sustainable competitive advantage of firms. In this paper, the mechanism of strategic orientation and business model innovation on corporate performance in a dynamic environment is investigated. Based on a sample of 356 Chinese construction firms, the partial least squares structural equation modelling (PLS-SEM) was adopted to test the hypotheses. It is found that entrepreneurial orientation and market orientation affect corporate performance differently. Unlike market orientation, which directly affects corporate performance, entrepreneurial orientation through business model innovation exerts influence instead of direct affection. Business model innovation plays a fully mediating role between entrepreneurial orientation and corporate performance and partially between market orientation and corporate performance. Meanwhile, environmental dynamism can positively moderate the relationship between business model innovation and corporate performance. This paper deepens the research on strategic orientation, business model innovation and corporate performance. The findings can provide a reference for construction firm managers to develop strategies and conduct business model innovation, which can finally help seek sustainable development in a dynamic environment.

## Introduction

In recent years, Chinese construction companies have effectively promoted the development of construction enterprises and industries by developing strategic orientation (Adams et al., 2019; Cake et al., 2020; Foltean and Glovatchi, 2021). On the one hand, taking the establishment of wholly-owned subsidiaries and project joint ventures with local enterprises as the entrepreneurial orientation, firms continuously cultivate emerging technologies, and expand enterprises’ scale. They have realized technological and institutional innovation and finally formed product competitive advantages (Wales et al., 2020). On the other hand, the market orientation provides excellent products or services and pays great attention to customer satisfaction (Ling et al., 2008). It requires improved market opportunity identification and development capabilities, expands existing markets, increases incremental markets, improves operational performance, and builds good customer service (Powers et al., 2020). However, with the intensified competition in the Chinese construction market and the impact of external factors such as the pandemic (Gu et al., 2020; Ren et al., 2022a), the continuous development of the Chinese construction industry has been dramatically threatened. According to the China Bureau of Statistics, the number of employees in China’s construction industry has decreased for three consecutive years. The profit margin of the construction industry has fallen for five consecutive years. In 2021 it even fell below 3 to 2.92%, the lowest in the last decade. Obviously, it is difficult for construction enterprises to obtain sustainable benefits and competitive advantages only by formulating differentiated strategies, and the role of strategic orientation in improving the performance of construction enterprises is facing new challenges (Stiles et al., 2021; Zhang et al., 2022).

The impact of strategic orientation on corporate performance has been widely concerned by scholars, but the mechanism of strategic orientation on corporate performance is controversial (Grimmer et al., 2017; Shih, 2018; Ali et al., 2020; Gotteland et al., 2020). Many scholars believe that strategic orientation can promote corporate performance (Atuahene-gima et al., 2006; Zhao et al., 2013; Tang et al., 2015; Oyewobi et al., 2016), especially entrepreneurial orientation and market orientation positively affect firm performance (Li et al., 2009; Aloulou, 2019). However, Song and Jing (2017) took China’s new ventures as the research object and found that entrepreneurial orientation positively impacts the performance of new ventures, while market orientation does not. This phenomenon shows that the impact mechanism of strategic orientation on enterprise performance is different. Changes in new scenarios will lead to changes in the relationship between strategic orientation and enterprise performance. Further, focusing on the practice of Chinese construction companies, we can find a significant gap between theory and practice regarding the relationship between strategic orientation and corporate performance. Does strategic orientation have a significant role in improving the performance of construction companies? Does increased environmental dynamics have an impact on this mechanism of action? In what ways does strategic orientation affect corporate performance? These are urgent problems to be solved with urgent theoretical and practical significance.

The strategic choice theory provides an appropriate theoretical perspective for this study. Strategic choice, by which managers determine the course of strategic action, involves the organization’s operating environment, economic constraints, and organizational structure design (Child, 1972). All strategic choices are to eliminate environmental constraints, corporate decision-makers and the external environment jointly decide strategic choices, the environment gives the agent corresponding spatial constraints, and the final decision of where the enterprise goes is attributed to the strategic choices made by the decision-makers (Child, 1997). Decision makers’ perceptions of the environment are shaped by their prior ideologies, and strategic actions are determined by actors’ and organizations’ prior cognitive frameworks, which exist in the form of embedded mindsets and cultures. The strategic choice theory argues that the forces and variables of the external environment are dynamic, and their interaction often influences business strategies, the effectiveness of organizational adaptation depends on organizational decision-making teams’ perceptions of environmental conditions and their decisions about how the organization should respond to those conditions (Judge et al., 2015). In order to eliminate environmental constraints, construction companies adopt business model innovation to maintain a sustainable competitive advantage in a dynamic environment. The categories range from business models themed on industrial parks, cultural tourism real estate, recreation centers, and TOD models to circular economy business models (Lee et al., 2016, 2017; Heesbeen and Prieto, 2020; Das et al., 2021; Gosselin et al., 2021). This paper argues that in a dynamic environment, companies adopt different strategic orientations to form a sustainable competitive advantage for the company (Spanjol et al., 2012; Cheng and Sheu, 2017; Han and Zhang, 2021). In which process business model innovation plays a vital role (Frank et al., 2019) because it can effectively reduce the transaction costs and plays an essential part in developing potential markets and improving the profitability of the corporate (Demil and Lecocq, 2010; Velu and Jacob, 2016; Visnjic et al., 2016).

Based on the above analysis, the questions studied in this paper are (1) Among construction firms, how does the strategic orientation affect corporate performance in a competitive and dynamic environment? (2) What role do business model innovation and environmental dynamism play in the relationship between strategic orientation and corporate performance? Following the logical evolution channel of “strategic orientation—innovative behavior—organizational performance” (Riviezzo et al., 2022), this paper divides strategic orientation into entrepreneurial orientation and market orientation (Nasir et al., 2017; Seet et al., 2021). Moreover, the mechanism of strategic orientation, business model innovation, corporate performance and environmental dynamism is explored. Thus, this paper has a dual purpose: confirmatory and predictive (Hair et al., 2019). Partial least squares structural equation modelling (PLS-SEM) is employed to test the hypotheses according to a sample of 356 Chinese construction firms. And a theoretical model of strategic orientation, business model innovation, and corporate performance is conducted. The findings can be helpful for construction firm managers to develop strategies and make business model innovations to achieve sustainable development in a dynamic environment.

The structure of the article is as follows: Section 2 summarizes the theoretical background of the article and proposes the research hypothesis. Section 3 introduces variable measurement methods, analyzes the and measurement scale validity, and introduces the research methods. Section 4 presents the results of the study. Section 5 summarizes the conclusions of the article, discusses the theoretical and practical value of this study, and briefly describes the main limitations of this study and some possible future research directions.

## Literature review and hypothesis

### Strategic orientation and corporate performance

Strategic orientation is a guideline for a company to achieve its strategic goals. It can fully reflect the company’s values and appears as a general understanding and cognitive interpretation of its external environment and internal resources (Selmi and Chaney, 2018). Strategic orientation reflects how a company operates and uses its resources, decision-making style, and approach (Barnett, 2008). The enterprise adopts a strategic orientation to achieve high performance based on the response and reshaping of the real environment. The strategy includes building new trading methods to meet potential market demands, rationally allocating resources to improve organizational efficiency (Zhang et al., 2020), and building high-efficiency trading networks to create user value to optimize and expand the industrial ecosystem (Eccles et al., 2014). On the other hand, corporate strategic orientation can reflect the underlying philosophical system, values, and corporate culture. It can reflect organizational strategies’ essential characteristics and attributes (Mu et al., 2017) and guide corporate decisions in a changing external environment (Lee and Chu, 2013). It has been shown that companies with a high market and technological orientation tend to have a high level of entrepreneurship. Entrepreneurial orientation with innovation, initiative and risk-taking characteristics is considered as the key to improving company performance (Aloulou, 2019).

This paper divides strategic orientation into market orientation and entrepreneurial orientation (Nasir et al., 2017; Seet et al., 2021). Narver et al. (2004) provide an in-depth analysis of the connotation of market orientation, divided into two dimensions: reactive market orientation and preemptive market orientation. Reactive market orientation focus on the existing market structure and the current needs of consumers. It discourages the proactive search for other opportunities to meet customers’ unperceived needs. In contrast, preemptive market orientation seeks to meet customers’ potential needs and emphasizes that firms should innovate their products by exploring the potential needs of consumers (Andreou et al., 2020; Bernoster et al., 2020). Innovativeness, risk-taking, and foresight are the core elements of entrepreneurial orientation. The key for entrepreneurial firms to gain a competitive advantage lies in uniquely and continuously innovating, even though risks accompany (Mishra, 2017). Above all, Entrepreneurial orientation allows firms to compete in the industry by taking risks, choosing innovation and making changes for competitive advantages. Organizations that implement entrepreneurial orientation in a dynamic competitive environment can better alter their way of business than those that do not (D'angelo and Presutti, 2019).

Corporate performance is an essential indicator of organizational success (Palacios-manzano et al., 2021). How construction firms can sustainably gain competitive advantages and improve corporate performance has become a vital issue (Shi et al., 2022). A study by Zott and Amit (2007, 2008) showed that the strategic choice of start-up firms has a crucial role in corporate performance improvement. The strategic orientation is socially complex, irreplaceable, reticent and practical. A precise strategic orientation can enhance companies’ competitive advantage through the rational allocation of resources. And it can drive the sprouting of new products, services and technologies. Also, it helps to bring a new paradigm for the organization to obtain success (Eccles et al., 2014). Market-oriented construction companies pay close attention to market information, such as customers, competitors, and internal and external environmental changes, and can quickly capture market information (Abbu and Gopalakrishna, 2021). Those fully interpreted and accumulated market knowledge effectively guide and motivate companies to make strategic behavioral choices and prompt them to build competitive advantages (Joshi, 2016; Tseng, 2016). Additionally, entrepreneurial construction business operators keep seeking new business opportunities for development (Helfat and Peteraf, 2015). They committed to competitive advantage acquisition and corporate performance improvement (Kollmann et al., 2017; Gao et al., 2018), tend to use their first-mover advantage to capture the market for the first time. And they take the initiative by establishing industry standards and occupying major distribution channels. Based on the above analysis, research hypotheses are proposed as follows.

*Hypothesis 1a*: Entrepreneurial orientation has a positive effect on corporate performance.

*Hypothesis 1b*: Market orientation has a positive effect on corporate performance.

### The mediating role of business model innovation

Business model innovation is defined as changing the corporate’s core elements and business logic (Bucherer et al., 2012). It means new organizational exchanges that can be achieved by connecting potential partners, offering new combinations of products, services, and information or designing new transaction mechanisms. Business model innovation includes innovation in additional products and services and generating new production methods, distribution or marketing (Zott and Amit, 2007; Zott et al., 2011; Amit and Zott, 2015). Teece (2007, 2018) considered business model innovation a component of dynamic capabilities. Different scholars classify business model innovation into different dimensions, including resource-driven innovation, product/service innovation, customer-driven innovation, financial-driven innovation (Osterwalder and Pigneur, 2013), product innovation, process innovation, and organizational innovation synergy. Among those classifications, Zott and Amit (2007) classification has been most adopted and widely accepted (Zott et al., 2011; Zott and Amit, 2013). Specifically, efficient business model innovation is based on transaction cost reduction and transaction efficiency improvement. Similarly, novel business model innovation involves transaction content and modality innovation (Cucculelli and Peruzzi, 2020). Novel business model innovation advocates that companies conduct economic transactions with transaction partners in new content or ways. It emphasizes new value propositions and new ways of transacting. Enterprises should make efforts to connect new transaction subjects in a broader range, adopt new ways to conduct transactions with various participants, design and improve new transactions and incentive mechanisms (Zott and Amit, 2007, 2008). Efficient business model innovation refers to implementing various activities by firms that can obtain transaction efficiency. This policy seeks to improve the current business model to reduce enterprise transaction costs. And it is by reducing the transaction complexity between enterprises and various participants, reducing information asymmetry between transaction activities and various stakeholders, and reducing errors in the transaction process (Amit and Zott, 2012).

The purpose of this paper is to show that strategic orientation has a catalytic effect on the business model innovation of construction companies. Specifically, market orientation is more reflected in the investment and analysis of the market (Ling et al., 2008). On the one hand, by accurately identifying customer needs and capturing the trend of consumption changes (Zhou and Park, 2020), considering existing resource combinations, and taking measures such as reducing costs or improving operational processes (Lin et al., 2021). Thus integration and optimal allocation of internal and external resources will be achieved effectively. Ultimately, it promotes the innovation of efficient business models (Arnold et al., 2011).On the other hand, by quickly collecting, processing, and understanding key market information, market orientation requires companies to analyze and predict market demand (Wales et al., 2020) and cooperate extensively with new partners to provide new product or service portfolios continuously (Chou and Yang, 2011). Also, it is oriented to promote novel business model innovation by mining and meeting market and consumer needs (Beck et al., 2011; Olofsson et al., 2018).

Entrepreneurial orientation can effectively integrate corporate resources, drive companies to acquire and respond to market information quickly, and seize innovation opportunities. And it leads firms to continuously design and develop new products and services that are unique, difficult to imitate and meet customer needs (Aloulou, 2019). In implementing of entrepreneurial orientation strategies, new markets, new technological knowledge, and the ability to provide solutions are needed (Ling et al., 2008). To create more value for customers and continuously enhance customer satisfaction and loyalty, novel business model innovation needs promotion (Gao et al., 2018). At the same time, entrepreneurial orientation can shape the perception of current or potential markets and their development trends, even further enhancing the ability to provide solutions. Utilizing and extending existing technologies and knowledge (Cheng and Huizingh, 2014) breaks through resource constraints in turbulent environments (Li et al., 2009; Song and Jing, 2017).

As a result, construction companies can continuously refine current transaction mechanisms and operational processes by adopting long-term differentiation strategies (Tang et al., 2007; Martek and Chen, 2016) and innovating business models (Chen et al., 2019) around technical quality, safety, and the environment. It is also conducive to enhancing the degree of market demand aggregation, improving transaction efficiency between partners, integrating and optimizing internal and external resources, and improving quality and customer service (Chang et al., 2018). Through the creation or improvement in construction technology, process and service forms (Chen et al., 2022), the innovation of the efficient business model will finally be realized (Powers et al., 2020). Therefore, this paper proposes hypotheses as follows.

*Hypothesis 2a*: Entrepreneurial orientation has a positive impact on business model innovation.

*Hypothesis 2b*: Market orientation has a positive influence on business model innovation.

At the same time, the efficiency-focused business model facilitates the exchange of information among participants. The accelerating information sharing speed gradually reduces the information asymmetry between partners, which is conducive to further aggregation of market demand, thereby greatly reducing transaction costs and promoting corporate performance improvement (Zott and Amit, 2008). In contrast, novel business model innovation focuses on exploring and satisfying market and consumer needs. Enterprises actively introduce new products or services and use the created market space to acquire more potential consumers, partners, and suppliers. Then through the effective optimal allocation of current resources to achieve wealth and income acquisition across organizational boundaries (Pucihar et al., 2019), enterprises can obtain value-added in the original market (Cortimiglia et al., 2016). On the one hand, developing and designing new transaction models and incentive models for businesses that can target customers in the original market and connect partners in a wider range is made easier by the innovation of novel business models (Shih, 2018). On the other hand, this business model can also better grasp customers’ purchase intention through new customer experiences and transaction methods. A new value creation process forms through these two aspects, and the value upgrade of potential resources in the existing market level attains (Ghezzi et al., 2015; Jang et al., 2019). This study proposes the following hypotheses.

*Hypothesis 3*: Business model innovation has a positive impact on corporate performance.

Based on the above analysis, market orientation is a business philosophy that can promote innovation in firms and make the innovation process rapidly updated and iterative, thus improving corporate performance (Aziz and Omar, 2013). Entrepreneurial orientation can make firms innovative, pre-emptive and risk-taking (Tseng et al., 2019). Also, it facilitates the implementation of business model innovation (Perez-luno et al., 2011). Besides, different types of firm strategy directly determine the firm’s ability to gain sufficient benefits from the business model innovation (Zott and Amit, 2008). In a word, market orientation and entrepreneurial orientation affect enterprises’ business model innovation process and then promote corporate performance (Saebi et al., 2017; Guo et al., 2020). Business model innovation plays an intermediary role between strategic orientation and corporate performance. Therefore, this paper proposes the following hypothesis.

*Hypothesis 4a*: Business model innovation plays a mediating role between entrepreneurial orientation and corporate performance.

*Hypothesis 4b*: Business model innovation plays a mediating role between market orientation and corporate performance.

### The moderating effect of environmental dynamism

Environmental dynamism refers to the rate at which the environment faced by the enterprise exhibits specific uncertainty and instability, and its focus is to indicate the change in the environment (Burgers and Covin, 2016; Ren et al., 2023). It is reflected in the volatility during the development process and the unpredictability of the final result. The main factors affecting environmental dynamism include possible environmental shocks, changes in industrial structure, and varying market demands (Ren et al., 2022a). Suppose the frequency, degree and unpredictability of changes are taken into account. In that case, both volatility (rate of change and amount of change) and unpredictability (uncertainty) are the essential characteristics of environmental dynamism (Zhang et al., 2020). Environmental dynamism can be elaborated through different dimensions, including market environment dynamics, policy environment dynamics, and technology environment dynamics (Tatarynowicz et al., 2016; Wang et al., 2022). Changes in customer composition and preferences cause market environment dynamics. Adjustments in economic policies and regulatory systems bring about policy environment dynamics. And technological advances bring about technology environment dynamics (Wirtz et al., 2010).

Environmental dynamism can facilitate the exchange of information to stimulate innovative behavior (Deng et al., 2021). However, there is no consensus on how environmental dynamism affects firm innovation and performance (Ren et al., 2022b). Commonly, environmental dynamism is divided into three main types. First, the role of environmental dynamism is negative between dynamic capabilities and corporate performance (Ringov, 2017). Second, environmental dynamism positively moderates the relationship between dynamic capabilities and corporate performance in a rapidly changing environment (Jiao et al., 2013; Karna et al., 2016; Andrade et al., 2021). Third, environmental dynamism positively moderates the relationship between dynamic capabilities and novel business model innovation and negatively moderates the relationship between dynamic capabilities and efficient business model innovation (Yuan et al., 2021).

The construction industry is widely regarded as dynamic due to the increasing uncertainty of technology, budget and development processes (Oyewobi et al., 2016; Zhang et al., 2021). As emerging technologies and international competition intensify, the highly competitive market environment becomes an unavoidable influence on the development of construction firms. Additionally, there are significant differences in corporate performance when the market environment varies (Zhao et al., 2012). Environmental dynamism positively moderates the relationship between green product innovation on firm cost performance and firm profitability (Chan H.K. et al., 2016). This paper argues that a higher level of environmental dynamism can force firms to absorb and utilize new information better, creating more new product configurations and products that are easier to transfer to new markets. Realize value creation through business model innovation (Sorescu et al., 2011), enhance the dynamic capabilities of construction enterprises (Li and Liu, 2014), and form a competitive advantage that is scalable (Dunford et al., 2010), difficult to imitate (Teece, 2010), and sustainable (Morris et al., 2005). Meanwhile, in a dynamic environment, business model innovation can weaken the uncertainty and complexity in transactions by allocating scarce resources and reducing coordination costs and transactions (Lee and Chu, 2013), ultimately improve operational efficiency and corporate performance (Zott and Amit, 2007, 2008). Once an enterprise makes the transaction cost decrease through the innovation of the business model, it will attract more new customers to participate in the transaction, thus bringing higher transaction volume and profit to the enterprise. Therefore, construction enterprises can create novel business model innovations for new products and services in a more dynamic environment, and adopt efficient business model innovation to reduce the transaction cost of transaction parties (Chan T. et al., 2016). In that case, the transaction efficiency of all parties can be improved to achieve the acquisition of competitive enterprise advantage and the improvement of corporate performance (Cooke et al., 2018). Based on the above discussion, this paper proposes the following hypothesis.

*Hypothesis 5*: Environmental dynamism positively moderates the relationship between business model innovation and corporate performance.

**Figure 1:**
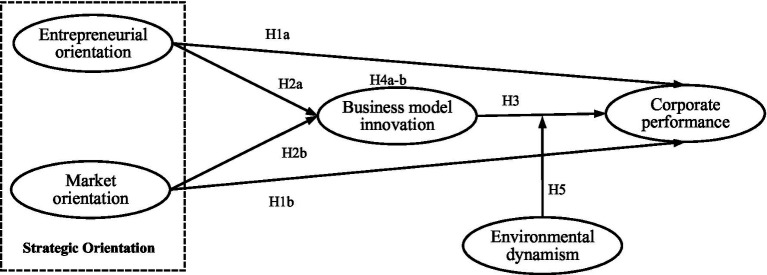
Research model.

To test the proposed hypothesis, a model that aims to investigate the direct impact of strategic orientation on corporate performance, as well as the mediating role of business model innovation and the moderating role of environmental dynamism is designed. Figure 1 presents the research model of this paper.

## Materials and methods

### Data collection and sample

The construction industry has an important position in China’s economic development. By the end of 2021, China had 128,746 construction enterprises, and the growth rate of the number of enterprise units reached 10.31%. A questionnaire was designed for this study, including two parts. The first part involves information about the respondents and the surveyed enterprises, including the gender, age, education level, as well as length of establishment and number of employees of the enterprises. The second part measured the strategic orientation, business model innovation, corporate performance and environmental dynamism of the surveyed companies by distributing questionnaires to managers of these companies. In order to reduce the influence of common method bias, the questionnaire adopts the method of anonymous measurement and cross-arrangement of items, hoping to improve the reliability of the data as much as possible (Palacios-manzano et al., 2021). Totally, we distributed 500 questionnaires and recovered 356 questionnaires effectively finished as the survey sample, with an effective recovery rate of 71.2%.

Descriptive statistics revealed that 75.28% of the respondents were male, and 24.72% were female. A 26–35 years old took up the most significant proportion, accounting for 56.18%. Most respondents had a bachelor’s degree, accounting for 46.91%, followed by a master’s degree, at 39.32%. The most significant number of respondents’ companies was established more than ten years, accounting for 64.04%. The number of employees in surveyed companies is 301–500, occupying 28.93%, followed by companies with more than 1,000 employees, at 25.28%.

### Measurement

In this paper, we adopt the established scales of existing scholars and improve them by taking into account the actual situation of Chinese construction enterprises. Strategic orientation is divided into entrepreneurial orientation and market orientation (Arnold et al., 2011; Grimmer et al., 2017). For entrepreneurial orientation, the scale of Jambulingam et al. (2005) is adopted to measure it. Six-question items are designed in terms of solid motivation, innovativeness and risk-taking ability. As for market orientation, this paper adopts Narver et al. (2004) definition and chooses 11 items to measure it. Meanwhile, drawing on relevant studies by Klassen and McLaughlin (1996) and Collins and Smith (2006), corporate performance is measured by four questions. Additionally, the scale of Zott and Amit (2008) was used to measure business model innovation with eight items. The scale developed by Jansen et al. (2006), Baron and Tang (2011), and Schilke (2014) was referenced to measure environmental dynamism through four-question items. The designed measurement scale was based on a five-point Likert scale, Figure 1 indicating disagree and 5 for very agree.

### Data analysis

PLS-SEM has been widely used in studies in strategic management (Hair et al., 2012; Wilden et al., 2013), marketing management (Hajli, 2015; Laato et al., 2020), and other fields. This study used PLS-SEM for data analysis. PLS-SEM is very suitable for this study. First, the statistical model of this study includes five composite variables, and PLS-SEM is very suitable for it (Cepeda-carrion et al., 2019). Second, PLS-SEM is a suitable technique for theory development, including mediating and moderating variables (Hashi and Stojcic, 2013). Third, PLS-SEM does not require a specific distribution and is valid for large or small samples (Willaby et al., 2015; Hair et al., 2017). While testing the hypothesis, a method based on 5,000 sub-samples was applied to ensure the stability of the results.

### Common method bias

This paper intends to test the endogeneity of the scale through VIF and Harman’s one-way variance. The results may have collinearity problems if the VIF is greater than 5 (Hair et al., 2019). The results show that the variance inflation factor (VIF) of the variables is all less than the threshold of 5 (Hair et al., 2012), and most of them are close to or less than 3 (Hair et al., 2017). We adopted Harman’s single-factor test and an unmeasured latent common method factor (ULCMF) to access common method variance (Bagozzi and Yi, 1990; Podsakoff et al., 2003). Harman’s single-factor test results showed that the five variables (KMO: 0.964; Bartlett sphericity test Sig. 0.000) had an explanation rate of 61.04%. The explanation rate of business model innovation (the main factor) is 35.334%, which is less than the critical value of 50%(Hair et al., 2017). According to the confirmatory factor analysis (CFA) results, the fit of the five-factor model (χ^2^/df = 2.873, CFI = 0.914, TLI = 0.906, RMSEA = 0.043) was significantly higher than that of the one-factor model (Δχ^2^ = 1585.71, Δdf = 43, *p* < 0.001). In addition, when comparing the five-factor model’s fitting index with that of the ULCMF measurement model (Δχ^2^/df = 0.608, ΔCFI = 0.032, ΔTLI = 0.031, ΔRMSEA = 0.013), the fitting indexes of the two differ slightly. Overall, the results show that the common method variance of the measurements is minimal. Table 1 shows the results of Common method bias analysis.

**Table 1:** Common method bias analysis.

Model	χ^2^	df	CFI	TLI	RMSEA
Five-factor model	1393.496	485	0.914	0.906	0.043
Single-factor model	2609.673	495	0.800	0.786	0.110
ULCMF	1023.960	452	0.946	0.937	0.030

## Results

Smart PLS 3 software was employed to analyze the PLS path model. With the research of Henseler et al. (2015, 2016) and Hair et al. (2017, 2021), the results’ interpretation comprises two stages: assessment of the measurement model and evaluation of the structural model.

### Measurement model

The model structure of this study was tested for reliability and validity, as shown in Table 2. Firstly, the factor loadings of most items in the five variables are greater than 0.7, which supports the reliability of the indicators. The factor loading of only one indicator is low. However, since the corresponding structure exhibits satisfactory internal consistency reliability and convergent validity, it follows the study of Henseler et al. (2016), keeping this indicator (Hair et al., 2021). Secondly, the Cronbach’s Alpha of all variables is higher than 0.8, which meets the interval that should be higher than 0.7. And the combined reliability CR value is higher than 0.8, which is greater than the minimum standard criterion of 0.6, indicating that the construction reliability of the scale is excellent (Hair et al., 2019). Third, the average variance extraction AVE is greater than 0.5, which supports the convergent validity of the scale construction measurement (Ali et al., 2016).

**Table 2:** The measurement model results.

	Item	Factor loading	*T*-value	α	CR	AVE
Corporate performance	CP1 compared with competitors in the industry, the corporation has better profitability	0.91	84.763	0.933	0.952	0.832
	CP2 compared with competitors in the industry, the corporation has higher profit margins	0.911	78.661						
	CP3 compared with competitors in the industry, the corporation has a higher market share	0.901	53.104						
	CP4 compared with competitors in the industry, the corporation has a higher sales growth rate	0.926	104.807						
Business model innovation	EBM1 the business model of the corporation can avoid errors in the transaction process as much as possible	0.818	33.043	0.951	0.959	0.746	
	EBM2 the business model of the corporation is highly applicable, able to handle large-scale or small-scale transaction activities	0.883	66.076							
	EBM3 the business model of the corporation can summarize the information, participants and services of the corporation in a larger area	0.88	58.395							
	EBM4 overall, our business model, can enable us to attain faster transaction efficiency	0.881	65.553							
	NBM1 the business model of the corporation can reintegrate the output services and products	0.884	60.924							
	NBM2 the corporation would use innovative incentive measures to increase the enthusiasm of business model participants	0.886	61.946							
	NBM3 the business model of the corporation has the most significant number of products or the most styled participants in history	0.824	33.453							
	NBM4 overall, our business model is novel	0.85	48.796						
Environment dynamism	ED1 the technology in this industry changes rapidly	0.878	56.793	0.844	0.920	0.742
	ED2 the market demand for this industry changes rapidly	0.837	33.513						
	ED3 the final product or service of this industry is updated quickly	0.88	48.211						
	ED4 the knowledge and skills required by the industry are updated rapidly	0.851	44.218						
Entrepreneurial orientation	EO1 the corporation encouraged and introduced innovative ideas, products, and services	0.805	31.034	0.858	0.896	0.593	
	EO2 the corporate leaders emphasized scientific research, technology leadership and innovation	0.796	29.715							
	EO3 the corporation strongly supported high-risk projects	0.536	9.669							
	EO4 the corporation agreed to take bold actions to achieve the set goals	0.745	22.177							
	EO5 the executives have introduced new ideas and products ahead of others	0.85	48.942							
	EO6 facing competitors, the corporation took the lead in introducing new products, services, management, and operation technologies	0.844	50.26						
Marketing orientation	MO1 we often analyzed and tracked customer needs	0.821	36.948	0.951	0.957	0.670	
	MO2 our corporation has valued customer satisfaction	0.764	29.361							
	MO3 gaining a competitive advantage has been based on the understanding of customer needs	0.832	40.582							
	MO4 our corporate strategy has aimed to create value for customers	0.786	32.39							
	MO5 our corporation has responded very quickly to the actions of competitors	0.845	43.913							
	MO6 our corporation’s business department usually shares competitive information	0.793	29.58							
	MO7 our corporation entered a market segmentation, allowing us to utilize our competitive advantages better	0.838	44.788							
	MO8 we regularly analyzed the advantages and disadvantages of corporations that produced similar products	0.864	58.901							
	MO9 our employees in all departments knew how to help customers create value	0.836	48.626							
	MO10 cooperation between various departments has been to achieve corporate goals	0.771	26.225							
	MO11 corporate managers knew how to explore the value of employees to meet customer needs	0.848	55.847						

Finally, the paper examined the discriminant validity between variables, as shown in Table 3. Each construct’s AVE should be compared to the squared inter-construct correlation (as a measure of shared variance) of that same construct, and to all other reflectively measured constructs in the structural model. Furthermore, the shared variance for all model constructs should not be larger than their AVEs (Hair et al., 2019). The correlations’ hetero-trait-single-trait (HTMT) ratios were all below the threshold of 0.90 (Voorhees et al., 2016), indicating the discriminative validity of the scale.

**Table 3:** Discriminant validity.

	BMI	CP	ED	EO	MO
BMI	**0.864**	0.847	0.648	0.847	0.899
CP	0.8	**0.912**	0.557	0.749	0.811
ED	0.596	0.508	**0.862**	0.658	0.624
EO	0.775	0.682	0.585	**0.77**	0.864
MO	0.858	0.769	0.573	0.765	**0.819**

HTMT ratio over the diagonal (italics). Fornell–Larcker criterion: square root of AVE in diagonal (bold) and construct correlations below the diagonal.

### Structural model evaluation

In this paper, we measure endogenous constructs’ *R*^2^ and *f*^2^ values as in-sample predictive power (Rigdon, 2012), and *R*^2^ values of 0.75, 0.50, and 0.25 can be considered substantial, moderate, and weak (Henseler et al., 2016), showing an *R*^2^ of 0.76 for business model innovation and 0.67 for corporate performance, indicating high explanatory power of the model (Shmueli and Koppius, 2011). Meanwhile, Q^2^, an indicator that combines out-of-sample predictive and in-sample explanatory power (Hair et al., 2019), yielded Q^2^ values much higher than zero (Q^2^_BMI_ = 0.563; Q^2^_CP_ = 0.550) for blindfolded results with an omission distance of 7, indicating high predictive accuracy of the constructed structural model (Hair et al., 2012).

### Hypothesis verification results

**Figure 2:**
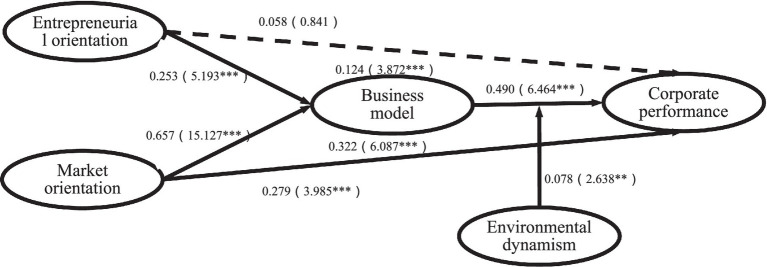
Path testing result. *p < 0.05, **p < 0.01 and ***p < 0.001.

The hypothesis verification results are shown in Table 4 and Figure 2. Among them, the path coefficient of entrepreneurial orientation and corporate performance is β = 0.058, and the *t*-value is 0.841. H1a does not hold. The path coefficient between market orientation and corporate performance is positive (β = 0.279, *t* = 3.985), indicating that market orientation positively impacts corporate performance, and H1b is established. The path coefficient of entrepreneurial orientation and business model innovation is β = 0.253, and the *t*-value is 5.193, indicating that entrepreneurial orientation positively impacts business model innovation, and H2a is established. The path coefficient of market orientation and business model innovation is β = 0.657, and the *t*-value is 15.127, indicating that entrepreneurial orientation positively impacts business model innovation, and H2b is established. The path coefficient of business model innovation to corporate performance is 0.49, which is significant at the 0.001 level, and H3 is established.

**Table 4:** Structural model and hypothesis verification.

	Path	*T*-value	f^2^	95CI	VIF	H	Supported
Direct effects							
EO → CP	0.058	0.841	0.003	[−0.08,0.196]	3.109	H1a	NO
MO → CP	0.279	3.985***	0.053	[0.140,0.414]	4.533	H1b	YES
EO → BMI	0.253	5.193***	0.098	[0.152,0.343]	2.716	H2a	YES
MO → BMI	0.657	15.127***	0.662	[0.575,0.743]	2.716	H2b	YES
BMI → CP	0.49	6.464***	0.170	[0.344,0.638]	4.334	H3	YES
Moderating effects	0.078	2.638**	0.021	[0.024,0.140]	1.031	H5	YES
Indirect effects							
EO → BMI → CP	0.124	3.872***		[0.068,0.195]		H4a	YES
MO → BMI → CP	0.322	6.087***		[0.225,0.430]		H4b	YES

**p < 0.01 and ***p < 0.001.

The results of moderating effect showed that environmental dynamism positively moderated the relationship between business model innovation and corporate performance (β = 0.078, *t* = 2.638, sig = 0.009). Thus, H5 was established. The mediating effect of business model innovation between strategic orientation and corporate performance was tested by bootstrapping. The results showed that business model innovation had a completely mediating effect between entrepreneurial orientation and corporate performance (β = 0.124, *t* = 3.872), and H4a was established. Business model innovation partially mediates market orientation and corporate performance (β = 0.322, *t* = 6.087), and H4b is established.

## Conclusion and discussion

### Conclusion

With accelerated market changes and frequent technological upgrades, construction companies face greater environmental dynamism and uncertainties, and it has become inevitable to re-examine their strategic positioning. Business model innovation has become an important way for enterprises to obtain value and maintain market competitiveness. In this context, this paper explores the intrinsic mechanism by which strategic orientation affects corporate performance. Based on a sample of 356 construction firms, the relationship between strategic orientation, business model innovation and corporate performance is investigated, and the moderating effect of environmental dynamism is explored. The strategic orientation in this paper includes two parts, “Market orientation” and “Entrepreneurial orientation.” The results show that both entrepreneurial orientation and market orientation positively impact business model innovation, and business model innovation also positively affects the performance of construction companies, which is consistent with the results of previous scholars (Bhatti et al., 2021; Klein et al., 2021). Specifically, firms that implement market orientation tend to be more likely to identify market opportunities, collect market information keenly, and innovate and adapt their business models according to customer needs and trends (Fang et al., 2014). Entrepreneurial orientation can often become a catalyst for enterprises to implement business model innovation. Implementing entrepreneurial orientation strategies can also improve business model innovation, drive enterprises to establish core competitive advantages, obtain customer value, and generate higher corporate performance (Jambulingam et al., 2005; Bernoster et al., 2020).

Entrepreneurial orientation and market orientation of construction enterprises have different mechanisms for influencing corporate performance. Companies use entrepreneurial-oriented influence mechanisms to take bold actions to achieve goals, improve products and services through business model innovation, and affect corporate performance. Different from market orientation, the entrepreneurial orientation of construction enterprises cannot directly promote corporate performance. This is maybe China’s economy has developed rapidly since China’s reform and opening up. Furthermore, massive investments in various industries have brought unprecedented market opportunities to the construction industry. However, in recent years, the central premise of the development of the construction industry is changing: the industry investment has reached its peak, and the market size has reached its peak; the high-end market is not so broad, and the mid-end market is shopping for costs, and the low-end market competition is disorderly and unprofitable. The competition in the construction industry is becoming more and more fierce. In this case, traditional businesses and products cannot bring sustainable competitiveness to enterprises. And construction enterprises can only formulate entrepreneurial orientation, followed by intensifying market competition. At the same time, in a dynamic environment, companies adopt entrepreneurial-oriented strategies with high risk, and forward-looking products and services cannot attract consumers to buy to generate performance directly (Lee and Chu, 2013). Business model innovation completely mediates between entrepreneurial orientation and corporate performance. And it partially mediates between market orientation and corporate performance. Market orientation aims at customer satisfaction and improving market influence, attaches importance to analyzing and tracking customer needs, and responds quickly to competitors’ actions to create value for customers. Therefore, market orientation can promote business model innovation and improve corporate performance (Amit and Zott, 2012).

Environmental dynamism positively moderates the relationship between business model innovation and corporate performance. Technology, market demand and market competition in the external environment are changing rapidly, products and services in the industry are rapidly updated (Bucherer et al., 2012; Ren et al., 2022c), knowledge and skills are rapidly iterated, and resources are increasingly difficult to obtain. The dynamic environment requires enterprises to adopt suitable business models to maintain their core competitive advantages (Amit and Zott, 2015). At the same time, the dynamic environment is an advantage for fast-growing enterprises because it forms specific business barriers, which can effectively avoid the excessive entry of potential entrants. Therefore, environmental dynamism can positively moderate the relationship between business model innovation and corporate performance (Jiao et al., 2013). In the face of a complex and highly dynamic market, enterprises need to rely on internal and external resources for business model innovation, which always carries risks. In this context, coping with and adapting to complex environmental dynamism has become a critical factor in determining whether business model innovation can improve corporate performance (Li and Liu, 2014). Business model innovation can not only promote the improvement of enterprise performance, but also actively and effectively cope with and adapt to the dynamic change of the environment.

### Theoretical contribution

This paper constructs a theoretical model of “strategic orientation - innovative behavior - organizational performance” through the main logic of “strategic orientation - innovative behavior - organizational performance” (Riviezzo et al., 2022). The theoretical contributions of this paper include the following three aspects:

First, this paper provides new theoretical evidence for the research on the relationship between strategic orientation and corporate performance, proposes and verifies the impact mechanism of strategic orientation on corporate performance, and enriches related research (Liu et al., 2013; Chang et al., 2018). The relationship between strategic orientation and corporate performance is controversial among scholars. This study takes Chinese construction companies as a sample to explore the relationship between strategic orientation and corporate performance. The results show that market orientation can improve firm performance in a dynamic environment, but entrepreneurial orientation cannot directly affect firm performance. This conclusion emphasizes that enterprises can no longer obtain performance directly by starting and developing new products and services in a fully competitive market environment (Li et al., 2008; Laforet, 2009). The mechanism by which entrepreneurial orientation affects enterprise performance has changed (Chou and Yang, 2011; Zhao et al., 2013).

Second, this paper further reveals the mediating role of business model innovation between strategic orientation and corporate performance, providing new ideas for related research (Zott and Amit, 2007). The research proves that business model innovation has a full mediating effect between entrepreneurial orientation and firm performance and a partial mediating effect between market orientation and firm performance. This conclusion shows that business model innovation can actively promote the development of construction enterprises (Zahra et al., 2006). In a dynamic and fully competitive environment, enterprises should actively innovate and improve the novelty and efficiency of business models to maintain their competitive advantages (Cucculelli and Peruzzi, 2020; Wales et al., 2020).

Third, this paper also enriches the related research on strategic choice theory. The strategic choice theory holds that corporate decision makers and the external environment jointly determine the choice of corporate strategy (Child, 1972, 1997). In a dynamic environment, construction companies formulate the entrepreneurial orientation of establishing wholly-owned subsidiaries and establishing project joint ventures with local companies, providing excellent products or services for market development, and paying close attention to customer satisfaction (Ling et al., 2008; Burgers and Covin, 2016). Therefore, it is necessary to adopt business model innovation to develop novel products and services. Because it can improve enterprises’ operating efficiency, save operating costs, and enhance their core competitiveness (Zott and Amit, 2007; Zott et al., 2011; Amit and Zott, 2015). It turns out that the more turbulent the external environment, the more companies should adopt business model innovation to help obtain high returns.

### Managerial inspiration

Construction enterprises must establish the correct strategic thinking in the fierce industry competition, identify opportunities in the dynamic environment, and obtain sustainable competitive advantages through business model innovation. This paper has the following implications for the management of construction enterprises:

Make strategic choices correctly in a dynamic environment. The external system’s polygon and business background have created higher enterprise requirements. Enterprises should pay attention to social and environmental factors ranging from the external economic environment, institutional policies, and cultural environment to laws and regulations, as well as industrial environmental factors such as industry development trends and technological innovation status. Thus, improving their sensitivity to environmental changes and ensuring good matches between their own strategic choices and the dynamics of the external environment. Moreover, striving to maintain its competitive advantage and ensure the excellent development of enterprise performance. At the same time, in selecting the senior management team, focus on the internal fit between team members, avoid the internal simplification of managers’ characteristics, and avoid the confusion of decision-making caused by information asymmetry, to help enterprises make relatively correct strategies.

Develop differentiated competitive strategies in an increasingly mature market environment. There are two critical points in formulating a differentiated competitive strategy to achieve business model innovation. Clarify the company’s market positioning and development vision. First, clarify the company’s market positioning and development vision. Enterprises must strengthen policy and market research, seize market-leading opportunities, and promote forward-looking corporate decision-making. The second is based on a solid corporate foundation. In the construction industry, companies with market advantages can seize the period of industry adjustment, strengthen resource integration, control the domestic market, and develop international markets. Small and medium-sized corporates in this industry must rely on mature enterprise development experience to quickly establish their core competitiveness.

Actively carry out business model innovation. Business model innovation guarantees that an enterprise maintains its competitive advantage. It can provide more profit space for the enterprise and promote it to formulate strategies. Those are beneficial to its development and conducive to the enterprise’s long-term development. Construction enterprises should combine their advantages to develop novel products and services to improve operational efficiency. While actively innovating business models, on the one hand, we should continue to build a culture of innovation and collaboration. Create a cultural atmosphere conducive to innovation, improve agile adaptability in constantly breaking traditional business models and reconstructing new business models, establish a value orientation for innovation, establish a fault-tolerant mechanism, and tolerate reasonable innovation failures. On the other hand, it is necessary to strengthen the training and incentives of innovative talents, varying from internal training mechanisms, the school-enterprise joint training mechanism, to cooperation with colleges and universities. Through targeted training, customized training, and school-enterprise joint training platforms, joint training is in line with the industry. Develop the talents needed, and stimulate the potential of professionals through equity incentives and development mechanisms, internal entrepreneurial platforms, partners and equity incentive models.

### Limitations and future research

This study still has some shortcomings and limitations that need to be improved in future research. First, the mediating variables between strategic orientation and firm performance need to be further explored. Some scholars have studied strategic flexibility, data capability, resource base and supply chain agility as intermediary variables. Thus the mechanism of strategic orientation on corporate performance still needs further research. Second, limited by the survey sample objects and the time nodes filled in, the survey results will be different if there are different groups of people and times. Further improvements are needed in the empirical method. Third, there are limitations in sample data. This study mainly focuses on the samples of Chinese construction enterprises. In future research, we hope to not only explore the boundary conditions of strategic orientation on firm performance from external factors (e.g., economic policy uncertainty), but also expand the sample collection to enhance the argument’s reliability further and deepen the existing conclusions of this study (Wang et al., 2022).

## Data availability statement

The raw data supporting the conclusions of this article will be made available by the authors, without undue reservation.

## Ethics statement

Ethical review and approval was not required for the study on human participants in accordance with the local legislation and institutional requirements. Written informed consent from the [patients/ participants or patients/participants legal guardian/next of kin] was not required to participate in this study in accordance with the national legislation and the institutional requirements.

## Author contributions

WH, YZ, and RL co-authored the thesis. WH was mainly responsible for the content of chapters 2, 3, and 4. YZ was responsible for the chapters 1 and 5, and writing and revising the full text. RL is mainly responsible for revising the full text, sorting out the logic of the paper, and making important contributions to the formation of this manuscript. All authors contributed to the article and approved the submitted version.

## Funding

This work was supported by the National Natural Science Foundation of China (72091311) and the National Natural Science Foundation of China (L2124026).

## Conflict of interest

The authors declare that the research was conducted in the absence of any commercial or financial relationships that could be construed as a potential conflict of interest.

## Publisher’s note

All claims expressed in this article are solely those of the authors and do not necessarily represent those of their affiliated organizations, or those of the publisher, the editors and the reviewers. Any product that may be evaluated in this article, or claim that may be made by its manufacturer, is not guaranteed or endorsed by the publisher.
